# Changes in prey selection and fitness of non-native *Erythroculter erythropterus* following estuarine restoration in the Nakdong River, South Korea

**DOI:** 10.1371/journal.pone.0328372

**Published:** 2025-09-24

**Authors:** Jeong-Soo Gim, Donghyun Hong, Dong-Kyun Kim, Maurice J.M. Lineman, Kwang-Seuk Jeong, Ji-Young Lee, Gea-Jae Joo, Hyunbin Jo

**Affiliations:** 1 BK21 FOUR Pusan National University Education and Research Center for Infrastructure of Smart Ocean City, Busan, Republic of Korea; 2 Department of Integrated Biological Science, Pusan National University, Busan, Korea; 3 K-water Research Institute, Daejeon, Republic of Korea; 4 International Centre for Water Security and Sustainable Management under the auspices of UNESCO, Hwaseong, Republic of Korea; 5 RCF Experimental School Taiyanggong North Street, Chaoyang District Beijing, P. R. China; 6 Department of Nursing Science, Busan Health University, Busan, Republic of Korea; 7 K-water, Ulsan office, Ulsan, Republic of Korea; 8 Department of Pet Health Care, Busan Health University, Busan, Republic of Korea; Central University of South Bihar, INDIA

## Abstract

Estuarine ecosystems globally are being restored through re-naturalization efforts, including the Nakdong River Estuary (NRE) in South Korea, which partially re-opened in 2019 after decades of disconnection. We investigated how this restoration affected the feeding ecology and fitness of *Erythroculter erythropterus* (Skygager), a non-native cyprinid that became dominant in the upper NRE following barrage construction. We surveyed fish populations, analyzed stomach contents using Next Generation Sequencing, and assessed morphological changes from May 2018 to June 2022. Following NRE opening, zooplankton and fish diversity indices increased, while phytoplankton and benthic invertebrate diversity decreased. *E. erythropterus* showed altered prey selection patterns, shifting from diverse prey items to predominantly zooplankton after NRE opening, coinciding with altered vertical migration patterns of plankton communities. While length distribution did not change significantly, the condition factor *K* decreased significantly, indicating reduced fitness. Body shape parameters also shifted toward more elongated forms, suggesting morphological responses to changed hydraulic conditions and feeding ecology. Our findings demonstrate that controlled estuarine reconnection can affect non-native species through cascading trophic effects, potentially serving both restoration and non-native species management objectives. This study highlights the complex ecological consequences of partial estuarine restoration and the importance of considering multiple trophic levels when evaluating restoration outcomes.

## 1. Introduction

Estuarine ecosystems represent unique environments where biological, physical, and chemical exchanges occur between marine and freshwater habitats [1]. Nutrient interactions between the two aquatic systems give rise to a diverse range of habitats within the estuarine environment [2,3]. These habitats are particularly important as breeding grounds for marine fishes, provide suitable environments for marine fish larvae, and offer protection from predators, acting as nursery and reproduction grounds, serving as refuges and feeding areas for fish, potentially optimizing survival, development, and growth [4,5]. However, over the past century, estuarine barrages have been constructed for a variety of purposes, including urbanization, tidal power generation, and traffic management [6], leading to the disappearance of many natural estuarine ecosystems [7].

With the increasing recognition of the importance of estuarine ecosystems, some governments have recently implemented policies focusing on the benefits of opening barrages to facilitate estuarine ecosystem restoration [8–10]. Restoration of estuarine ecosystem permits saltwater inflow from the marine ecosystem, which considerably alters the local biota [11–13] by impacting; (i) morphological traits (such as brook trout gills and stickleback caudal peduncles and head size, and Cyprinidae fish from pectoral fin to ventral fin; [14–16]); (ii) migration patterns (such as fish’s habitat selection, move to feeding area and hatching area selection; [17–20]); (iii) life-history characteristics (such as fish’s maximum body size and fitness of small pelagic fish; [21–24]); (iv) community structure (such as decreasing stability of fish community and impact of non-native species; [25–27]); (v) behavioural patterns (such as European sea bass diet and feeding response of Cyprinidae fish; [28,29]). Several studies have examined the effects of environmental changes on morphological traits and migration patterns, but gaps remain in understanding how these changes affect community structure, behavioural patterns, and life history characteristics [30]. Furthermore, existing studies have principally focused on examining the effects of individual factors, which limits understanding sequential results generated by cascading effects for a series of processes.

Prior to the construction of the Nakdong River Estuary (NRE) barrage in 1987, the system functioned as a dynamic natural estuary with seasonal variations in river flow and salinity gradients [31]. During the monsoon season (summer), increased freshwater input pushed the saltwater boundary downstream, while during dry seasons (winter), saltwater extended further upstream. This natural variability created diverse microhabitats supporting rich biodiversity, including important commercial fish species and benthic communities adapted to fluctuating salinity conditions [31]. The natural NRE provided essential ecological functions including nutrient cycling, sediment transport, and connectivity between riverine and marine environments. In South Korea, the construction of the NRE barrage in 1987 severely disrupted the river-sea connectivity [31]. The barrage was initially constructed to secure freshwater supplies and prevent saltwater intrusion, but it effectively transformed the estuarine ecosystem into a primarily freshwater system. This transformation was further intensified by the Four Major Rivers Project, which deepened water channels and further decreased habitat diversity [32]. These changes had profound ecological consequences, including a sharp decrease in native species diversity [31,33] and the proliferation of non-native freshwater species [34]. The altered hydrological conditions created lentic (still water) habitats favoring non-native species adapted to lake-like conditions. Recognizing these ecological impacts, the Korean government initiated a restoration effort through partial re-opening of the NRE barrage, creating a brackish transition zone to potentially recover species diversity [35]. This controlled re-opening provides a valuable opportunity to explore the ecological consequences of partial estuarine restoration [36].

*Erythroculter erythropterus* (skygager) is a cyprinid fish species native to China, Russia, and parts of Korea, but considered non-native to the Nakdong River basin [37]. This predatory fish typically inhabits lentic water bodies such as lakes and reservoirs, preferring slow-flowing or still water environments [38]. *E. erythropterus* is primarily zooplanktivorous in its juvenile stages but becomes increasingly piscivorous as it matures, feeding on small fish and large zooplankton [39]. The species has become dominant in the upper reaches of the NRE following barrage construction, likely due to the creation of reservoir-like conditions that favor its ecological requirements [33]. Recent studies have documented the spatial distribution and migration patterns of *E. erythropterus* in the Nakdong River basin, including its responses to water quality variations [40]. Understanding how this dominant non-native species responds to estuarine restoration efforts is crucial for managing both the species and the overall ecosystem.

Optimal Foraging Theory (OFT) provides a theoretical framework for understanding predator feeding decisions based on energy maximization principles [41,42]. According to OFT, predators should preferentially select prey that maximize their net energy intake, considering both the energy gained from consumption and the costs associated with searching, capturing, and handling prey [43]. The concept of prey profitability – defined as the ratio of energy gained to handling time – is central to OFT predictions [42]. When multiple prey types are available, predators are expected to exhibit prey switching behavior, shifting their preference toward more abundant or profitable prey types to optimize their foraging efficiency [44,45]. In aquatic ecosystems, changes in environmental conditions can alter prey availability and profitability, potentially leading to shifts in predator selectivity patterns. The re-establishment of estuarine conditions in the NRE likely altered the relative abundance and accessibility of different prey types for *E. erythropterus*, providing an opportunity to examine how environmental restoration affects predator-prey dynamics through the lens of optimal foraging principles.

In this study, we investigated how the partial restoration of estuarine conditions resulting from barrage opening affected the food sources and fitness (specifically condition factor *K* and body shape parameters) of *E. erythropterus*, a dominant non-native species in the upper NRE. Specifically, we (1) compared differences in potential food sources and stomach contents of *E. erythropterus* using Next Generation Sequencing (NGS) analysis, and (2) explored changes in the fitness and morphological characteristics of *E. erythropterus* before and after NRE opening. We hypothesized that the shift from predominantly freshwater to partial estuarine conditions would alter available food sources, which would subsequently affect the prey selection patterns and fitness of this non-native species. Understanding these responses may provide insights into how controlled barrage management could be used as a potential tool for managing non-native fish populations while promoting ecosystem restoration.

### 1.1. Study area and NRE re-opening protocol

**Fig 1:**
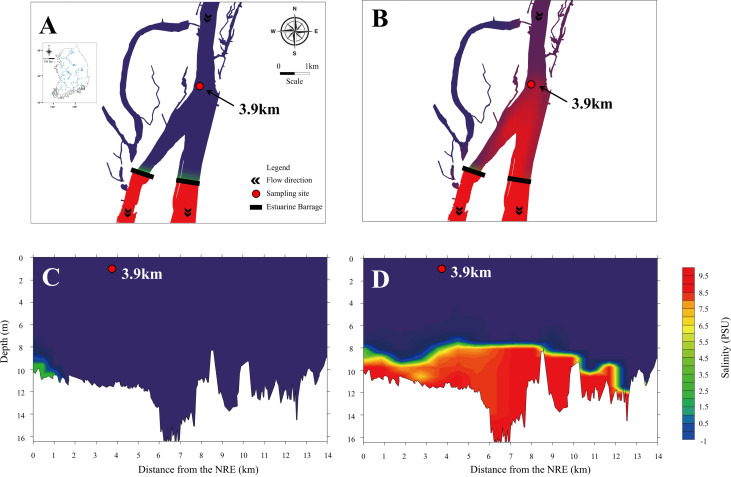
Description of the study sites in the NRE. Appearance after intrusion of different amounts of seawater before opening the NRE (A: Sky view; C: Cross-section view) and after intrusion of 9,300,000 tons (third opening of the NRE) of seawater (B: Sky view; D: Cross-section view). The amount of salt is represented by colours ranging from blue (0) to red (greater than 9.5).

The Nakdong River is the longest river in South Korea, with a length of 510 km and a drainage area of approximately 23,384 km^2^. Rainfall occurs primarily during summer due to the monsoon, but in winter it is low. Therefore, storing fresh water is important [46]. The Nakdong River barrage (35°08′20.3″N, 128°57′26.2″E) was constructed from 1983 to 1987 to serve as a sustainable water supply and to control saltwater intrusion [47]. The barrage comprising 10 sluices, has four regulatory sluices (length: 47.5 m, height: 8.3 m) and six main gates (length: 47.5 m, height: 9.2 m). The regulatory sluices release freshwater during the rainy season (typically July–August) and at low tides, thus maintaining the water level. Conversely, the main gates typically remain closed (Fig 1A, C, and S1 Fig).

When the opening began, only one regulatory gate (gate number 9) was utilized (Fig 1A, C, and S1 Fig). The regulatory gate was opened by dropping the lower (S1 Fig) or raising the upper (S1 Fig) halves of the gate. This gate was opened seven times between 2019 and 2022 (S1 Table in S1 File). The first and second openings served as pilot runs, during which approximately 1 million tons of saltwater flowed in less than 1 hour following the opening (S2 Table in S1 File). Beginning with the third opening of the NRE, the opening period was extended to approximately one month, during which the highest recorded saltwater flow occurred (9.3 million tons [47.8% of total amount of seawater intruded]; Fig 1B, D; S2 Table in S1 File). Therefore, our study period was divided into before and after the third opening of NRE (2020-06-04), which recorded the highest salt inflow.

## 2. Materials and methods

### 2.1. Field survey and data collection

To assess the impact of opening the NRE, we chose a study site located 3.9 km upstream, based on the extent of salinity penetration before and after the NRE opening (Fig 1A). This study site has the representativeness of monitoring salt inflow from both branches of the Nakdong River in the upper part of the NRE, which divides into two branches and flows to the sea (Fig 1A). Fish sampling was carried out at the study site from May 2018 to June 2022 (Fig 1; 10 in total; 35°07′35.00″N, 128°57′11.37″E). Fishes were collected for 24 h during each sampling period using a fixed shore net (mesh: 15 × 15 mm, height: 1 m, leader net: 15 m) to which local fishermen attached three fish traps (each mesh: 15 × 15 mm, width 1.5 m; height 1.8 m; length 3 m). Collected fish were identified and classified according to Kim and Park [37] and Nelson [48] and then released (S3 Table in S1 File). However, exotic species were not released, as this is prohibited by the act on protecting native and endemic species (Act on the Conservation and Use of Biological Diversity, Act No. 14513, Dec.27, 2016 by Ministry of the Environment). Morphological parameters (total length ± 0.1 cm, weight ± 0.1 g) of *E. erythropterus*, exotic species from other basins in South Korea, were measured immediately following capture, and their stomachs were removed for stomach content analysis. To avoid contamination from foreign-derived DNA, we clamped the lower oesophageal portion using forceps and dissected the stomach using medical scissors rinsed in 98% methanol and flame-sterilised. The stomach and tissue samples were preserved in 99% ethanol and separated into prey items. All samples were stored at room temperature prior to the analysis. All fish sampling and handling procedures were conducted in accordance with ethical guidelines and with approval from the Pusan National University Laboratory Safety Management Center (Institutional Animal Care and Use Committee). We obtained field research permission (No. 1380) from the National Institute of Environmental Research. Exotic species were not released back to the river as prohibited by the Act on the Conservation and Use of Biological Diversity (Act No. 14513, Dec. 27, 2016) by the Ministry of Environment. Fish were euthanized humanely using an overdose of MS-222 (tricaine methanesulfonate) prior to dissection and tissue sampling to minimize suffering.

We measured water quality parameters including water temperature, dissolved oxygen (DO), pH, conductivity, and salinity for surface water. Furthermore, we collected water samples (2 L at a depth of 0.5 m) from the fish sampling sites to analyse total nitrogen (TN) and total phosphorus (TP) levels before (20 May 2020) and after (4 June 2020) opening the NRE. A DO meter (YSI Model 58, USA) was used to measure water temperature and DO; conductivity and pH were measured using a conductivity meter (YSI model 152; Yellow Springs Instruments, Yellow Springs, OH, USA) and a pH meter (Orion Model 250A; Orion Research Inc., Boston, MA, USA), respectively. The TN and TP concentrations were determined using a spectrophotometer (Japan Fantec Research Institute, Shizuoka, Japan) as described by Wetzel and Likens [49]. To evaluate the maximum saltwater inflow after opening the NRE, we collected data on saltwater inflow quantities at 1 km intervals based on previous literature [50].

For phytoplankton, zooplankton, and benthic invertebrate analyses, we followed standardized sampling and identification protocols. Phytoplankton samples were collected using a Van Dorn water sampler at 0.5 m depth, preserved with Lugol’s solution, and concentrated by sedimentation. Identification was performed using an Olympus BX51 microscope at 400 × magnification following taxonomic keys by [51]. Cell counts were performed using a Sedgwick-Rafter counting chamber, and biomass was estimated using standardized biovolume measurements and conversion factors [52]. Zooplankton were collected by vertical hauls using a conical net (mesh size 64 μm, mouth diameter 30 cm) from near-bottom to surface. Samples were preserved in 4% formaldehyde solution and later identified to species level using identification keys [52]. Counting was conducted using a Bogorov counting chamber under a stereomicroscope (Olympus SZX16). Benthic invertebrates were collected using a Petersen grab sampler (0.025 m^2^) with three replicates at each sampling site. Samples were sieved through a 500 μm mesh, preserved in 70% ethanol, and sorted under a stereomicroscope. Specimens were identified to the lowest taxonomic level possible using appropriate taxonomic keys [53]. Biomass was determined as wet weight after blotting.

### 2.2. Potential food source before and after the NRE opening

To evaluate potential food sources for *E. erythropterus*, we compared the diversity indices of phytoplankton, zooplankton, benthic invertebrates and fish before and after NRE opening. The dominant and subdominant species were classified based on the species with highest relative abundance. For phytoplankton, zooplankton and benthic invertebrates, the results of the literature search were used [50], and the diversity indices of fish were calculated using the results of our field survey. The dominance [54], diversity [55], evenness [56], and richness [57] indices were calculated. These indices were computed using PAST 3.0 [58] based on data for the number of individuals. To assess vertical diel migration patterns, we conducted stratified sampling at three depths (surface: 0.5 m, middle: 6 m, and bottom: 10 m) before (10 May 2022) and after (23 May 2022) NRE opening. At each depth, water samples (2 L) were collected using a Van Dorn water sampler for phytoplankton, and zooplankton were collected using a horizontal tow of a 64 μm mesh net at each depth stratum. All samples were collected during daylight hours (10:00–14:00) to standardize potential diurnal effects. Abundance was calculated as individuals per liter for each depth, and vertical distribution patterns before and after NRE opening were compared by examining proportional changes at each depth. Due to the limited sampling occasions (one sampling event before and after opening), descriptive comparisons rather than statistical tests were used to assess changes in vertical distribution patterns (Fig 6).

### 2.3. Stomach contents of *E. erythropterus* before and after the NRE opening

To achieve high-resolution identification of prey items in fish stomach contents, we employed Next Generation Sequencing (NGS) for amplifying prey DNA, which provides superior taxonomic resolution and accuracy compared to traditional visual examination methods [59]. To analyze prey selection patterns and compare food sources before and after NRE opening, we randomly selected 24 *E. erythropterus* (before the NRE opening (12); and after the NRE opening (12). Stomach contents and tissue samples were extracted, stored separately in a fresh state, and rinsed in autoclaved water to avoid external and self-DNA contamination. Ethanol was completely volatilised from the samples prior to DNA extraction. The samples were then homogenised. Genomic DNA from each stomach sample was isolated using the DNeasy Blood and Tissue Kit (Qiagen) according to the manufacturer’s protocol. We selected the V9 region of the 18S rDNA gene (18S V9), primarily because of its broad range in eukaryotes [60]. NGS approaches using the 18S V9 region have recently enabled accurate metabarcoding for diet characterization across diverse taxonomic groups [61], making it an established approach for biomarker analysis [62]. The library preparation followed standard protocols with modifications for stomach content samples as detailed in the supporting Information appendix.

We sequenced library samples using NGS and identified all species taxonomically. The library was sequenced using an Illumina iSeq platform (Illumina, San Diego, CA, USA) and data processing was performed using USEARCH (v11.0.667) [63]. Demultiplexed raw sequences (FASTQ files) were merged into one sequence, allowing for a maximum of ten mismatches. Merged reads with expected errors >3.0 were discarded after filtering for quality. The remaining sequences were de-replicated and clustered into operational taxonomic units (OTUs) at a 97% OTU cutoff value, removing chimeric and singleton sequences. In total, 542,207 filtered paired-end reads from 24 samples were generated using the platform, of which 97% passed the Q30 (Phred quality score >30). Each sample yielded 10,044–29,397 paired-end reads (mean: 20,903 reads) and rarefaction curve analysis indicated that the number of OTUs were saturated for all samples (S2 Fig). Merged reads were processed and clustered into OTUs using the UCLUST [64], at 97% OTU cut-off value (153 OTUs in gamma diversity). The resulting 57 OTUs were classified into 11 genus-level taxonomic groups (S4 Table in S1 File).

The resulting out sequences were searched against the NCBI database (Release 250.0; July 2022) using BLASTn [63]. We obtained a list of the top 153 taxa for each OTU with the highest identity percentage. Considering the known characteristics and distribution of each taxon, we excluded taxa not present at the study sites. If all taxa found in the search results had low scores (query cover<80%, identity <85%) or were regarded as not present, the OTUs were deleted. Finally, the taxa with the highest identity was assigned to each OTU. OTUs with 97% or higher identity were identified at the species level, while the rest (93–97%) were identified at the gene level. OTUs identified as *E. erythropterus* were considered ‘self-DNA’ and excluded from the subsequent procedure. Obtained sequences were deposited in the NCBI repository under accession number PRJNA935053.

We constructed a phylogenetic tree based on known or empirical predator-prey relationships among taxonomic groups. Species traits of fish, aquatic insects, and protozoa were retrieved from the literature and database repositories [65]. All species or genera were classified into one of eight taxonomic groups: etc (Unionicolidae, worms, and protozoa), fish, fungi, amoeba, phytoplankton, zooplankton, magnoliophyta, or cnidaria. To compare opening-mediated spatiotemporal changes in the NRE, we divided prey items before the NRE opening group and after the NRE opening group. A detailed description of the phylogenetic construction protocol is provided in the methods section of the appendix.

**Fig 2:**
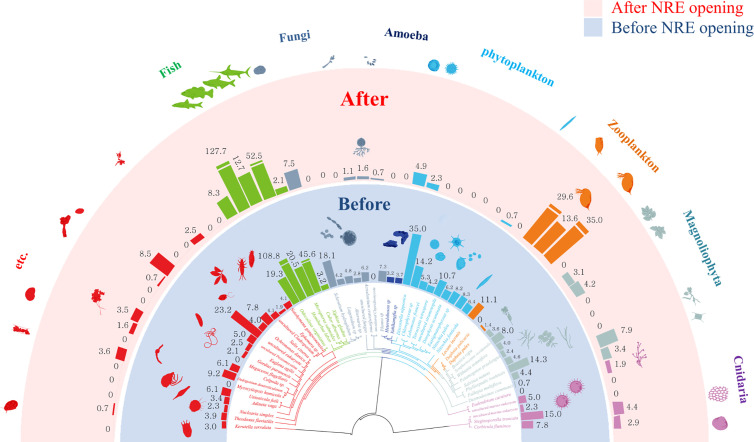
NGS datasets recovered from stomach contents of *E. erythropterus* in the NRE. A taxonomic phylogenetic tree of prey items identified from fish stomach contents was constructed, with bar graphs showing the operational taxonomic unit (OTU) count per phylum or order. Silhouettes represent the prey organism types; when a food source appeared both before and after the NRE opening, its silhouette was placed outside the circular bar. Red and blue colors represent prey items found after and before NRE opening, respectively.

To evaluate prey selection patterns, we compared the relative abundance of potential prey items in the environment with their occurrence in *E. erythropterus* stomach contents. Environmental prey abundance data were obtained from diversity analyses of phytoplankton, zooplankton, benthic invertebrates, and fish communities (Table 2), while stomach content data were derived from NGS analysis results (Fig 2). Prey selection was assessed qualitatively by comparing proportional representation between environmental availability and stomach contents before and after NRE opening, following established approaches for analyzing predator feeding selectivity in the presence of multiple prey types [43,44].

To compare patterns of preferred food sources before and after NRE opening, a self-organising map (SOM) analysis was performed. SOM is an algorithm that reduces data dimensionality [66]. The Kohonen network is a competitive network system where neurones in the Euclidean map space compete against each other. SOM training was achieved using a series of species data obtained from *E. erythropterus* stomach contents. A detailed description of the SOM modelling analysis is provided in the methods section of the appendix. A total of 24 data samples (before NRE opening (12); and after NRE opening (12)) were collected for each variable. No strict rules regarding the number of output neurones that should be obtained were present [67]; therefore, we trained the SOM with different map sizes to choose a suitable model. A batch learning algorithm was used in this study. After training, data were clustered according to the calculated U-matrix, which provided the dissimilarity of data samples on the map plane. Clustering mostly focused on organizing feeding patterns into clusters based on (i) different discrete series of species in the stomach and (ii) before and after NRE opening. The SOM model was developed using MATLAB 6.1 with the SOM Toolbox [68]. Model validation was performed using quantization error and topographic error metrics to ensure proper network training (S3 Fig). The final neighborhood size and learning rate were determined through iterative optimization to minimize these error values while maintaining interpretable output clusters. Complete SOM mapping results for individual species patterns are provided in S4 Fig.

### 2.4. Population structure and fitness of *E. erythropterus* before and after the NRE opening

During the study period, a total of 309 length-weight data points were obtained for *E. erythropterus*. The analysis was based on two groups of data: before NRE opening (n = 205, May 2018 to May 2020) and after NRE opening (n = 104, June 2020 to June 2021). Sampling effort (equipment, duration, and methodology) was identical during both periods, with the difference in sample sizes reflecting actual changes in capture rates. This difference in sample size was accounted for in statistical analyses through appropriate normalization procedures. To exclude seasonal effects from the sampling periods (pre and post opening), we conducted a field survey during the same season (May and June). We verified the significance of the distribution of total fish length before and after opening the NRE. The distribution of total *E. erythropterus* length was categorised, and significance was confirmed using chi-square distance analysis. In our feature selection task for the two-class problem, we computed the chi-square distance using the following equation [69]:


$$\begin{array}{c} \sum_{\text{i}=\text{1}}^{\text{n}}\frac{{\left({\text{x}}_{\text{i}}-{\text{y}}_{\text{i}}\right)}^{\text{2}}}{\left({\text{x}}_{\text{i}}+{\text{y}}_{\text{i}}\right)} \end{array}$$ (1)

where *n* is the number of bins, xi is the value of first bin and *y*_i_ is the value of the second bin. This was a simple and quick approach to ranking histogram bins and was selected because it was suitable for examining distributional changes when comparing two or more clusters [69].

Length-weight relationships (LWRs) assessing the fitness of *E. erythropterus*, were used to estimate the weight correspondent to a given length, whereas condition factors were employed to compare fish condition, considering that heavy fish of a given length were in good condition [70]. The LWRs of each species were estimated based on the equation:


log W=log a+b log L (2)

where log *a* is the regression intercept and *b* is the regression slope [71]. Prior to regression, outliers were removed using linear regression of the log-transformed equation [70]. The condition factor (*K*) was estimated from the relationship *K* = 100 *W*/*L*^3^ to assess the fish conditions [70]. Additionally, the form factor (a) was used to determine whether the body shape changed significantly, with reference ranges for different body forms: eel-like (0.00099–0.00165), elongated (0.00775–0.00906), fusiform (0.01310–0.01400), and short and deep (0.01720–0.01930) [70].

## 3. Results

### 3.1. Changes in water quality and potential food sources following NRE opening

Surface water quality variables (water temperature, DO, TN, TP, pH, conductivity and salinity) showed minimal differences before and after opening the NRE (Table 1). However, a pronounced vertical stratification was observed after NRE opening, with salinity of water samples collected from ≥ 8 m depth reaching ≥ 5 psu compared to only 0.4 psu for the surface water (Fig 1D, S2 Table in S1 File). This vertical salinity gradient was not present before the NRE opening.

**Table 1:** Comparison of water quality parameters in the surface water before and after Nakdong River Estuary (NRE) opening. DO: dissolved oxygen; TN: total nitrogen; TP: total phosphorus.

Water quality	Before	After
Water temperature (°C)	20.2	24.3
DO (mg/L)	8.2	9.1
TN (mg/L)	2.295	2.042
TP (mg/L)	0.030	0.028
pH	7.5	7.8
Conductivity (μS/cm)	338	339
Salinity	0.1	0.1

NRE opening did not result in a change in dominant phytoplankton species, but substantially increased the relative abundance of the dominant species *Aulacoseira ambigua f. japonica* from 18.0% to 67.5% (Table 2), representing a 3.75-fold increase. The dominance index more than doubled after NRE opening (from 0.14 to 0.38), while the diversity, evenness, and richness indices all decreased (Table 2), with diversity showing the most notable decline (from 2.41 to 1.73).

**Table 2:** Comparison of ecological indices of potential prey communities for *E. erythropterus* in the NRE before and after barrage opening. RA: Relative Abundance; ind.: number of individuals.

Ecological indices	Phytoplankton		Zooplankton		Benthic Invertebrates		Fish	
	Before	After	Before	After	Before	After	Before	After
Dominant species (RA, % / biomass or abundance)	*Aulacoseira ambigua f. japonica* (18.0 / 1,320,840)	*Aulacoseira ambigua f. japonica* (67.5 / 5,862,375)	*Daphnia* *longispina* (37.8 / 134)	*Daphnia* *longispina* (9.0 / 50)	*Limnodrilus* *gotoi* (22.4 / 268)	*Limnodrilus* *gotoi* (25.7 / 308)	*Erythroculter erythropterus* (93.5 / 186)	*Erythroculter erythropterus* (78.3 / 112)
Subdominant species (RA, % / biomass or abundance)	*Oscillatoria* spp. (16.5 / 1,210,770)	*Oscillatoria* spp. (4.7 / 408,195)	*Mesocyclops leuckarti* (4.4 / 16)	*Diaphanosoma brachyuru* (8.8 / 40)	*Grandidierella japonica* (11.4 / 137)	*Chironomidae indet.* (22.7 / 272)	*Lepomis macrochirus* (5.5 / 11)	*Lepomis macrochirus* (15.4 / 22)
Dominance	0.14	0.38	0.70	0.77	0.67	0.24	0.88	0.49
Diversity	2.41	1.73	1.68	1.75	1.33	0.39	0.28	1.14
Evenness	0.60	0.44	0.70	0.78	0.74	0.56	0.33	0.39
Richness	3.50	3.30	1.70	1.44	1.30	0.32	0.57	1.82

In zooplankton communities, NRE opening resulted in a shift in subdominant species from *Mesocyclops leuckarti* to *Diaphanosoma brachyuru* (Table 2). The dominant species remained *Daphnia longispina*, but its relative abundance decreased markedly from 37.8% to 9.0%, representing a 76% reduction. The zooplankton richness index decreased from 1.70 to 1.44, while the dominance, diversity, and evenness indices all showed increasing trends (Table 2).

For benthic invertebrates, the dominant species remained *Limnodrilus gotoi* both before and after NRE opening, with a slight increase in relative abundance (from 22.4% to 25.7%). Following re-opening, a notable shift in subdominant species occurred from the estuarine amphipod *Grandidierella japonica* to predominantly freshwater Chironomidae larvae, with relative abundance of the latter increasing from 11.4% to 22.7%, representing almost a two-fold increase. This shift appears contradictory to expectations as *G. japonica* is typically associated with estuarine conditions while Chironomidae are primarily freshwater organisms. This unexpected result may be related to local habitat modifications or short-term disturbance effects following the opening. All benthic invertebrate diversity indices (dominance, diversity, evenness, and richness) decreased following NRE opening (Table 2), with the richness index showing the most dramatic decline (from 1.30 to 0.32), a 75% reduction.

*E. erythropterus* (a non-native species) and *L. macrochirus* (another non-native species) were the dominant and subdominant fish species before NRE opening, respectively, constituting 93.5% and 5.5% of total abundance. After NRE opening, these species remained dominant, but the relative abundance of *E. erythropterus* decreased from 93.5% to 78.3% (Table 2), a 16% reduction indicating a potential restructuring of the fish community. The fish community dominance index decreased substantially after opening the NRE (from 0.88 to 0.49), while diversity, evenness, and richness indices all increased notably (Table 2), with the richness index showing the most dramatic improvement (from 0.57 to 1.82), representing a 3.2-fold increase. These changes in fish community structure suggest that the partial restoration of estuarine conditions may reduce the dominance of non-native freshwater species, potentially providing more opportunities for native estuarine species.

### 3.2. Shifts in prey diversity and feeding patterns of *E. erythropterus* following NRE opening

The total number of OTUs in the stomachs decreased significantly from 52 (91.2% of total identified OTUs) to 29 (50.9%) after NRE opening (χ² = 146.7, df = 114, *p* = 0.021), indicating a substantial reduction in prey diversity as measured by OTU richness (Fig 2, S4 Table in S1 File). Food source distribution revealed that the overall proportions of fungi, phytoplankton, and other species decreased after opening the NRE, while the overall proportion of zooplankton increased (Fig 2). In phytoplankton, a variety of species were distributed before the NRE opening; however, following NRE opening only *Discostella* sp. (freshwater species), *Chlorophyceae* sp. (marine species), and *Nitzschia* sp. (marine species) were present (Fig 2). For zooplankton, Daphnia spp. (freshwater species) and *Lecane* sp. (freshwater species) showed a significant increase in distribution proportions after NRE opening (Fig 2). With respect to fish species, since the re-opening of the NRE, the proportion of Cyprinidae increased, while the proportion of Perchidae decreased (Fig 2).

**Fig 3:**
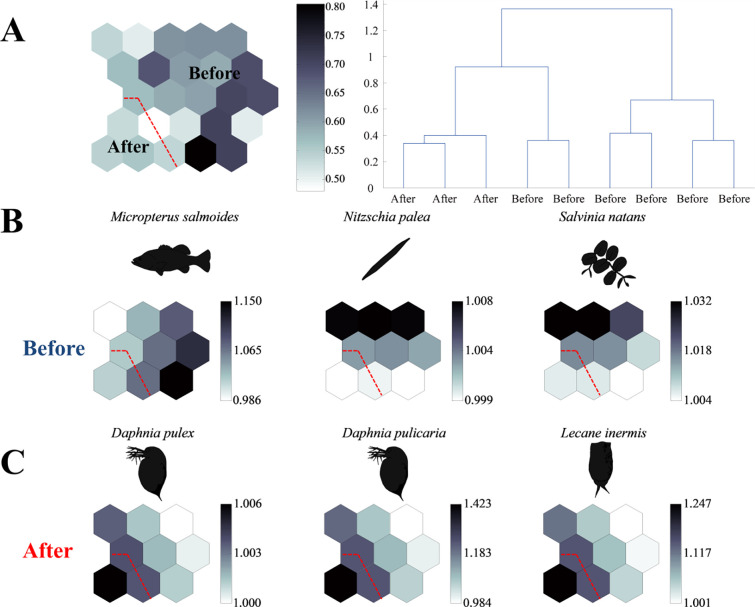
The results of self-organising map (SOM) clustering. (A) U-matrix clustering and dendrogram showing dissimilarity of the cells on the map plane. The patterning results for proportion of OTU numbers per phylum or order parameters (B) before and (C) after the NRE opening. Colour scale is related to distances between map units. Black colours represent large distances and white colours represent small distances. The diagonal red line separates the two main clusters (before and after NRE opening) identified by the SOM analysis.

The Self-Organizing Map (SOM) analysis of *E. erythropterus* stomach content data revealed distinct dietary patterns before and after opening the NRE (Fig 3). The SOM model differentiated feeding patterns into two clearly distinct clusters based on prey composition. Using a dissimilarity threshold value of 1.0 as the clustering criterion, nine cells on the map plane were diagonally grouped according to the U-matrix (quantisation error, 1.502; topographic error, 0.000), resulting in clear separation between samples collected after NRE opening (lower left) and before NRE opening (upper right) segments (Fig 3A). For prey items with the highest aggregation rates, three distinct groups were identified in each period (Fig 3B and C). Before opening the NRE, prey item distribution was diverse, with relatively even representation of fish, phytoplankton, and other prey groups (Fig 3B); however, after opening the NRE, zooplankton (particularly *Daphnia* spp. and *Lecane* sp.) became notably more dominant in the diet (Fig 3C). This pronounced shift in diet composition indicates a major change in feeding behavior following the altered estuarine conditions.

To assess prey selection patterns, we compared the relative abundance of prey items in the environment (Table 2) with their occurrence in *E. erythropterus* stomach contents (Fig 2). Before NRE opening, zooplankton comprised 37.8% of environmental abundance but showed lower representation in stomach contents, suggesting selective feeding on other prey types including fish and phytoplankton. After NRE opening, despite zooplankton environmental abundance decreasing to 9.0%, they became the dominant prey in stomach contents, indicating a shift from selective feeding to opportunistic feeding on the most accessible prey. This pattern suggests that *E. erythropterus* prey selection was influenced more by prey accessibility and capture efficiency than by environmental abundance alone, particularly following the altered vertical distribution of zooplankton toward surface waters after NRE opening.

### 3.3. Effects of NRE opening on population structure and fitness of *E. erythropterus*

**Fig 4:**
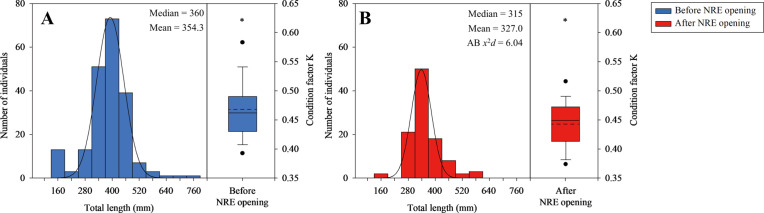
Length distribution and condition factors *K* of *E. erythropterus* (A) before and (B) after the NRE opening. Before, 205 individuals; After, 104 individuals.; A statistical significance of the *t*-test (*P* < 0.05) performed to deduce if there is significant difference between condition factors K before and after opening the NRE; Solid line: median; Dash line: mean; * *P* < 0.01.

**Fig 5:**
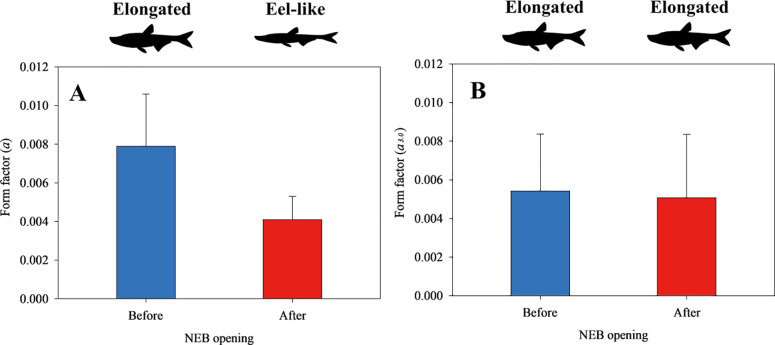
Form factor of *E. erythropterus* (A) and standardized form factor *a*_3.0_ (B) before and after opening the NRE. Before, 205 individuals; After, 104 individuals. Range of form factor for body shape categories: Elongated (0.00775–0.00906), Eel-like (0.00099–0.00165). Error bars represent standard deviation.

The chi-squared distance analysis revealed differences in population size structure before and after NRE opening (χ^2^ distance = 6.04) (Fig 4), indicating a notable change in size distribution. *E. erythropterus* total length was normally distributed, ranging between 150–800 mm before NRE opening and 150–600 mm after opening (Fig 4A and B). The most notable change was the complete absence of very large individuals (>600 mm) after NRE opening, despite identical sampling effort. Additionally, the proportion of medium-sized individuals (300–450 mm) decreased substantially after NRE opening, with the number dropping from 73 individuals before opening to only 18 individuals after opening, representing a 75% reduction. This shift in size structure suggests potential changes in population demographics following altered estuarine conditions. We compared the length-weight relationship (LWR) parameters and condition factor *K* (as indicators of fitness) before and after NRE opening (Table 3). The b value (the slope of LWR; Eq. 2) increased from 2.97 to 3.07, while the *a* value (intercept of LWR; Eq. 2) decreased from 0.0079 to 0.0041 after NRE opening. The non-overlapping 95% confidence intervals for the *a* value (0.0052 ~ 0.0120 vs. 0.0029 ~ 0.0058) suggest a meaningful change in body shape toward a more elongated form following NRE opening. Most importantly, the mean condition factor *K* after NRE opening decreased significantly compared to pre-opening conditions (from 0.47 to 0.43; *t* = 3.809, df = 292, *P* < 0.001; Fig 4), indica*t*ing reduced overall fitness of *E. erythropterus* following the re-establishment of estuarine conditions. This change occurred despite the differences in size distribution, suggesting that the reduced condition factor reflects actual changes in fish fitness rather than merely being an artifact of different size compositions. The change in body shape was further confirmed by analyzing the form factor (*a*), which showed a significant decrease from 0.0079 before NRE opening to 0.0041 after opening (Fig 5A). This change indicates a shift from the ‘elongated’ form category (0.00775–0.00906) towards the ‘eel-like’ form category (0.00099–0.00165). When standardized to a common slope (*a*_3.0_), the form factor still showed a decrease, though less pronounced (Fig 5B). These morphological changes are consistent with the observed changes in length-weight relationship parameters and further support the finding that *E. erythropterus* underwent notable physical adaptations following changes in environmental conditions.

**Table 3:** Descriptive statistics and estimated parameters of length-weight relationships (w = *a*L^*b*^) for *E. erythropterus* before and after opening the NRE.

NRE opening	n	*a*	95% CL of *a*	*b*	95% CL of *b*	*R* ^2^
Before	205	0.0079	0.0052 ~ 0.0120	2.97	2.77 ~ 3.00	0.96
After	104	0.0041	0.0029 ~ 0.0058	3.07	2.97 ~ 3.17	0.96

*a*, intercept; *b*, slope; *R*^2^, coefficient of determination.

### 3.4. Vertical distribution patterns of plankton before and after NRE opening

**Fig 6:**
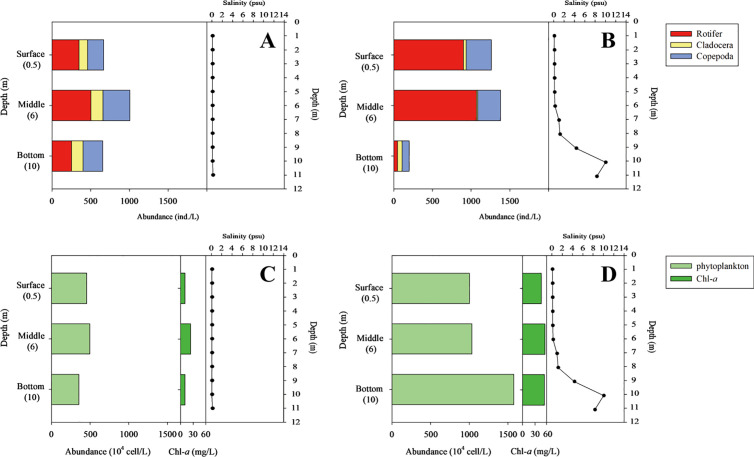
Distribution of zooplankton abundance by depth (A) before and (B) after and phytoplankton abundance by depth (C) before and (D) after the opening of the NRE. Sampling period: before NRE opening (10 May 2022); after NRE opening (23 May 2022).

Following NRE opening, notable changes were observed in the vertical distribution of both zooplankton and phytoplankton (Fig 6). Zooplankton showed a marked shift in vertical distribution patterns. Before NRE opening, zooplankton abundance was relatively evenly distributed across depths, with slightly higher concentrations in middle waters. After opening, zooplankton abundance increased dramatically in surface (0.5 m) waters, with rotifer abundance increasing 2.6-fold (from 350 to 899 ind./L) and copepod abundance increasing 1.6-fold (from 205 to 324 ind./L). Conversely, zooplankton abundance decreased substantially in bottom waters (10 m), with rotifer abundance decreasing by 82% (from 254 to 46 ind./L) and cladoceran abundance decreasing by 58% (from 146 to 61 ind./L) (Fig 6A, B). Phytoplankton showed an opposite pattern, with higher concentrations developing in bottom waters after NRE opening (Fig 6C, D). Before opening, phytoplankton were relatively evenly distributed throughout the water column, but after opening, their abundance increased by 44% in bottom waters (from 3,542,500–5,100,000 cells/L) while decreasing by 78% in surface waters (from 4,562,500–1,001,750 cells/L). These changes in vertical distribution patterns coincided with the development of a strong salinity gradient, with higher salinity (≥ 5 psu) in bottom waters after NRE opening. The vertical redistribution of plankton communities, particularly the shift of zooplankton toward surface waters, likely increased their availability as prey for surface-feeding fish like *E. erythropterus*.

## 4. Discussion

This study compared changes in food sources and fitness of the non-native cyprinid *E. erythropterus* before and after partial re-opening of the Nakdong River Estuary. *E. erythropterus*, native to parts of northeastern Asia but introduced to the Nakdong River, typically inhabits lentic (still water) environments and is predominantly zooplanktivorous when young, becoming increasingly piscivorous with age [39]. In the NRE system, this species became dominant following barrage construction which transformed the estuary into a more reservoir-like environment. Our results revealed a significant reduction in food item diversity and an altered prey selection from a diverse diet to mainly zooplankton following the re-establishment of partial estuarine conditions. *E. erythropterus* trophically is a high-level predator in aquatic ecosystems [39], while the food sources identified from gastric contents for fish from our study sites mainly consisted of organisms from lower-trophic-levels (Fig 2). This finding was similar to other studies, which reported that changes in low-trophic-level communities’ diversity influenced by dam construction ultimately impacted high-trophic-level fish populations [23,72]. In addition, saltwater influx appears to be a key factor affecting the fitness of high-trophic-level consumers through both direct physiological stress and indirect effects via food web alterations. The apparent reduction in fitness of *E. erythropterus* following NRE opening likely reflects this species’ preference for freshwater environments over brackish conditions.

From an optimal foraging perspective, the observed dietary shift in *E. erythropterus* following NRE opening can be interpreted as a response to changes in prey profitability rather than simply prey availability. While zooplankton abundance increased after restoration, the shift toward predominantly zooplankton consumption may reflect suboptimal foraging conditions. According to OFT principles, the profitability of prey items depends not only on their energy content but also on capture efficiency and handling time [41,43]. Fish prey typically provide higher energy returns per individual compared to zooplankton, making them more profitable despite lower abundance [44]. The observed reduction in condition factor *K* suggests that the increased reliance on zooplankton, while energetically feasible, was less efficient than the previous more diverse diet. This finding aligns with OFT predictions that predators forced to rely on less profitable prey due to environmental constraints will exhibit reduced fitness outcomes [42]. The vertical redistribution of plankton communities following salinity stratification likely influenced prey encounter rates and capture success, further affecting the cost-benefit ratio of different foraging strategies. The concentration of zooplankton in surface waters may have increased their detectability but also their escape probability, altering the overall profitability of zooplankton as prey.

The vertical redistribution of plankton communities [73–75] we observed after NRE opening likely influenced prey availability for *E. erythropterus*. The upward migration of zooplankton toward surface waters in response to bottom water salinization appears to have made them more accessible to *E. erythropterus*, which typically feeds in surface to mid-water depths [40,39]. This behavioral response by zooplankton to avoid increased salinity in bottom waters is consistent with previous studies documenting salinity avoidance behaviors in freshwater zooplankton [76–78]. However, despite increased availability of zooplankton in the feeding zone of *E. erythropterus*, their fitness decreased, suggesting that the dietary shift toward almost exclusive zooplankton consumption was suboptimal for this species. This could be due to energetic constraints, as zooplankton generally provide less energy per individual prey item compared to fish prey [79,80].

The comparison between environmental prey availability and stomach contents revealed important insights into *E. erythropterus* prey selection strategies. Before NRE opening, this species demonstrated selective feeding behavior, consuming fish and diverse prey types despite zooplankton being the most abundant prey in the environment (37.8% relative abundance). This selective behavior aligns with optimal foraging theory predictions, where predators choose prey that maximize energy returns relative to handling costs [43]. Fish prey, though less abundant, provide higher energy content per individual compared to zooplankton, making them more profitable prey items [81,82]. Following NRE opening, the feeding strategy shifted dramatically. Despite zooplankton environmental abundance decreasing to 9.0%, they became the predominant prey in stomach contents. This apparent contradiction can be explained by the altered spatial distribution of prey following salinity stratification. The upward migration of zooplankton to surface waters increased their encounter rates with *E. erythropterus*, effectively increasing their availability despite lower overall abundance [43,44]. This shift represents a transition from energy-maximizing selective feeding to accessibility-driven opportunistic feeding, consistent with optimal foraging theory when preferred prey become less accessible [45]. The observed reduction in condition factor *K* following this dietary shift supports the interpretation that increased reliance on zooplankton, while energetically feasible, was less efficient than the previous more selective diet. This finding demonstrates how environmental changes can force predators to adopt suboptimal foraging strategies, ultimately affecting their fitness [43,45].

The observed changes in the fish community following NRE opening, particularly the decreased relative abundance of *E. erythropterus*, suggest potential shifts in competitive and predatory interactions (Figs 3 and 4). The increased fish community diversity indices after NRE opening indicate that other fish species, potentially including native estuarine species, may have gained competitive advantages under the new environmental conditions [81]. Furthermore, the complete absence of larger *E. erythropterus* individuals (>600 mm) after NRE opening could indicate either size-specific emigration from the study area or increased predation pressure on larger individuals by piscivorous predators that may have gained access to upper reaches following reconnection [82]. While this study focused primarily on *E. erythropterus* as a dominant species, these community-level interactions likely played an important role in the observed responses and warrant further investigation.

Therefore, it is possible that zooplankton, based on their increased abundance in the surface water, could increase their probability of becoming a food source for *E. erythropterus*. The food source and mouth shape of *E. erythropterus* suggest that they feed on organisms in surface waters or middle depths [39,40]. Subsequently, our findings suggest that this effect could propagate from zooplankton (low-trophic-level consumers compared to fish) to *E. erythropterus* that prey on them. These changes in food sources could affect the fitness of fish, as observed in studies on the reduction for condition factors of salmon due to changes in feeding behaviour resulting from variations in prey availability [83]. These food sources therefore changed alter competition and predation pressure, which in turn might drive fitness of *E. erythropterus* [84].

The observed changes in zooplankton diversity following NRE opening may reflect complex interactions between environmental changes and predation pressure. While *E. erythropterus* increased its consumption of zooplankton after restoration, the zooplankton diversity indices (evenness and richness) showed increasing trends (Table 2). This apparent paradox can be explained by the reduced overall abundance and condition of *E. erythropterus* following NRE opening, which likely decreased total predation pressure on zooplankton communities despite increased per-capita consumption. Additionally, the altered vertical distribution of zooplankton may have created spatial refugia in deeper waters with higher salinity, reducing their vulnerability to surface-feeding predators. These findings highlight the importance of considering both direct predation effects and indirect environmental modifications when assessing top-down control in aquatic ecosystems [45]. The complex interplay between predator fitness, prey accessibility, and environmental heterogeneity demonstrates how restoration efforts can trigger cascading effects throughout the food web.

Even though our results didn’t show significant differences for the population structure of *E. erythropterus* (Fig 4), the condition factor *K* (fitness) decreased significantly after the NRE opening (Fig 4). Also, our results which show that a shift in the food source from a broad range of prey items (Fig 3B) to a narrower range, focused on zooplankton (Fig 3C), resulting in a decrease in the fitness of individuals that consumed the altered food source (Fig 4). Crowding low-trophic-level communities might result in temporary reductions in the fitness of high-trophic-level communities due to concentrated food sources [84]. Based on our results, it was found that food source diversity like benthic invertebrates and phytoplankton decreased, while the diversity of zooplankton increased after NRE opening (Fig 2, Table 2). This implied a shift in food source structure. In particular, zooplankton, the main food source for *E. erythropterus*, have a low carbon content, which may adversely affect *E. erythropterus* condition, unlike the fish food sources they had previously consumed [79,80].

Fish undergo sensory adaptations and morphological shifts in response to changes in environmental connectivity and food availability [85,86]. Our findings indicate a significant decrease in the length-weight relationship parameter a, representing a shift in body form from a deeper-bodied elongated shape toward a more eel-like form (Table 3, Fig 6). This morphological plasticity likely represents an adaptive response to altered flow regimes and prey selection behavior.

More elongated body forms are generally associated with more efficient swimming in flowing water environments [87], suggesting that *E. erythropterus* may be adapting to the increased water movement following NRE opening. Additionally, the shift toward zooplankton as a primary food source may favor different foraging strategies requiring morphological adjustments. While such morphological plasticity typically develops over multiple generations, the relatively rapid changes observed here suggest phenotypic plasticity within the existing population. Similar rapid morphological responses have been documented in other cyprinid species following habitat alterations [88–90]. Additionally, plastic shifts, like phenotypic plasticity due to fish life-history traits, involve reduced lifespans and maximum body sizes as well as increased reproductive efforts after impoundment [23,88–91]. We suspected that plastic shifts of *E. erythropterus* might be influenced by re-opening the NRE. Therefore, our study showed that NRE re-opening can produce negative effects on food source shifts and fitness for *E. erythropterus*.

An important consideration for interpreting these results is the potential influence of seasonal variations in physico-chemical parameters and biological communities between monsoon and non-monsoon periods [92]. While our sampling was designed to control for seasonal effects by conducting surveys during the same seasons (May and June), the NRE system likely experiences significant seasonal fluctuations that interact with the effects of barrage opening [93]. During monsoon seasons, increased freshwater input would push the saltwater boundary downstream, potentially reducing the estuarine influence even when the barrage is partially open [94]. Conversely, during dry periods, saltwater intrusion would extend further upstream, potentially increasing stress on freshwater-adapted species like *E. erythropterus* [95]. These seasonal dynamics may significantly influence both the short-term responses we observed and the long-term ecological trajectory of the system following restoration efforts.

The dominance of non-native species such as *E. erythropterus* in the Nakdong River system [33] represents a significant management challenge, as these species can reduce native biodiversity and alter ecosystem functioning [96]. Our findings suggest that partial barrage opening could serve as a potential management tool for controlling non-native freshwater species while promoting estuarine restoration. The observed reduction in *E. erythropterus* fitness and the concurrent increase in overall fish diversity indices indicate that controlled estuarine reconnection may help shift the ecosystem toward a more natural state. Various approaches to estuarine restoration have been implemented globally, ranging from complete barrier removal [97,98] to various degrees of managed connectivity [99–101]. While complete removal would maximize ecological connectivity, it would eliminate the water management benefits provided by the barrage. Our results suggest that a middle-ground approach using controlled, partial openings may provide ecological benefits while maintaining water management capabilities. The management implications extend beyond *E. erythropterus* to the broader ecosystem. The increased fish diversity following NRE opening suggests a potential pathway for native species recovery, though longer-term monitoring would be needed to confirm this trend. However, it is important to recognize that management decisions should not be based solely on the response of a single species, even if dominant. Rather, a holistic ecosystem approach considering multiple trophic levels and native species recovery should guide barrage management strategies [102,103].

## 5. Conclusions

Our study provided insights into the ecological consequences of partial estuarine restoration through controlled barrage opening in the Nakdong River Estuary. We observed a significant shift in the preferred food source of the non-native *E. erythropterus* from a diverse diet to primarily zooplankton following NRE opening, accompanied by reduced fitness as measured by condition factor *K*. These changes coincided with altered vertical distribution patterns of plankton communities and significant changes in community structure across multiple trophic levels. While several studies have recommended complete barrier removal to fully restore original estuarine conditions [97,98,104], our findings highlight the complex ecological responses that can occur during partial restoration. The apparent negative effects on *E. erythropterus* fitness, while potentially beneficial from an invasive species management perspective, illustrate how rapid environmental changes can create challenges for established populations. Our results align with previous studies showing that morphological traits and food web structures can be significantly impacted by altered habitats following restoration efforts [105,106]. Future research should investigate the long-term responses of the NRE ecosystem to different opening regimes, considering variables such as opening duration, timing relative to seasonal cycles, and the number of open sluice gates. Additionally, expanding monitoring to include a broader range of native and non-native species would provide a more comprehensive understanding of community-level responses. We emphasize that barrage management should be approached with caution, as sudden environmental changes require organisms to adapt rapidly, potentially causing temporary stress even in species that might ultimately benefit from restoration. In conclusion, while our study focused primarily on a single dominant non-native species, the observed cascading effects—from changes in plankton communities to fish fitness—demonstrate the interconnected nature of estuarine food webs. These findings suggest that controlled barrage opening could potentially serve both ecological restoration goals and non-native species management objectives, but a gradual, adaptive management approach would likely provide the best pathway toward sustainable estuarine restoration.

## Supporting information

S1 Fig Diverse regulatory gate forms. (A) The regulatory gate remains closed, blocking freshwater and seawater mixing. (B) The underflow and (C) the overflow method for opening the regulatory gate.
(TIF)

S2 Fig Rarefaction curves of in *Erythroculter erythropterus*’s stomach contents in NGS results. (A to X: Sample 1 to Sample 24).
(TIF)

S3 Fig The results of SOM clustering. (A) Clustering of the cells on the map plane, (B) dendrogram showing dissimilarity of the cells in the map, (C) Clustering of the cells on the map plane by three groups and (D) The patterning results for before and after NRE opening on the SOM plane, B: Before NRE opening; A: After NRE opening.
(TIF)

S4 Fig The patterning results for stomach of *Erythroculter erythropterus* on the SOM plane. Colour scale is related to distances between map units. Black colours represent large distances and white colours represent small distances.
(TIF)

S1 File **S1 Table. Specific information of opening experiments conducted. S2 Table. Salinity (psu) distribution at the time of the maximum seawater inflow in the NRE. S3 Table. Fish assemblage in the NRE (2018 ~ 2022).** ▲, exotic species; #, introduced species; ※ endemic species. **S4 Table. Identified taxa in the stomach contents of *Erythroculter erythropterus* before (n = 12) and after (n = 12) the opening of the NRE.**
(ZIP)
