# Supplementary figures and images for: Changes in prey selection and fitness of non-native Erythroculter erythropterus following estuarine restoration in the Nakdong River, South Korea
Source: PLoS One. 2025 Sep 24;20(9):e0328372. doi: 10.1371/journal.pone.0328372 (PMC12459770; doi:10.1371/journal.pone.0328372)

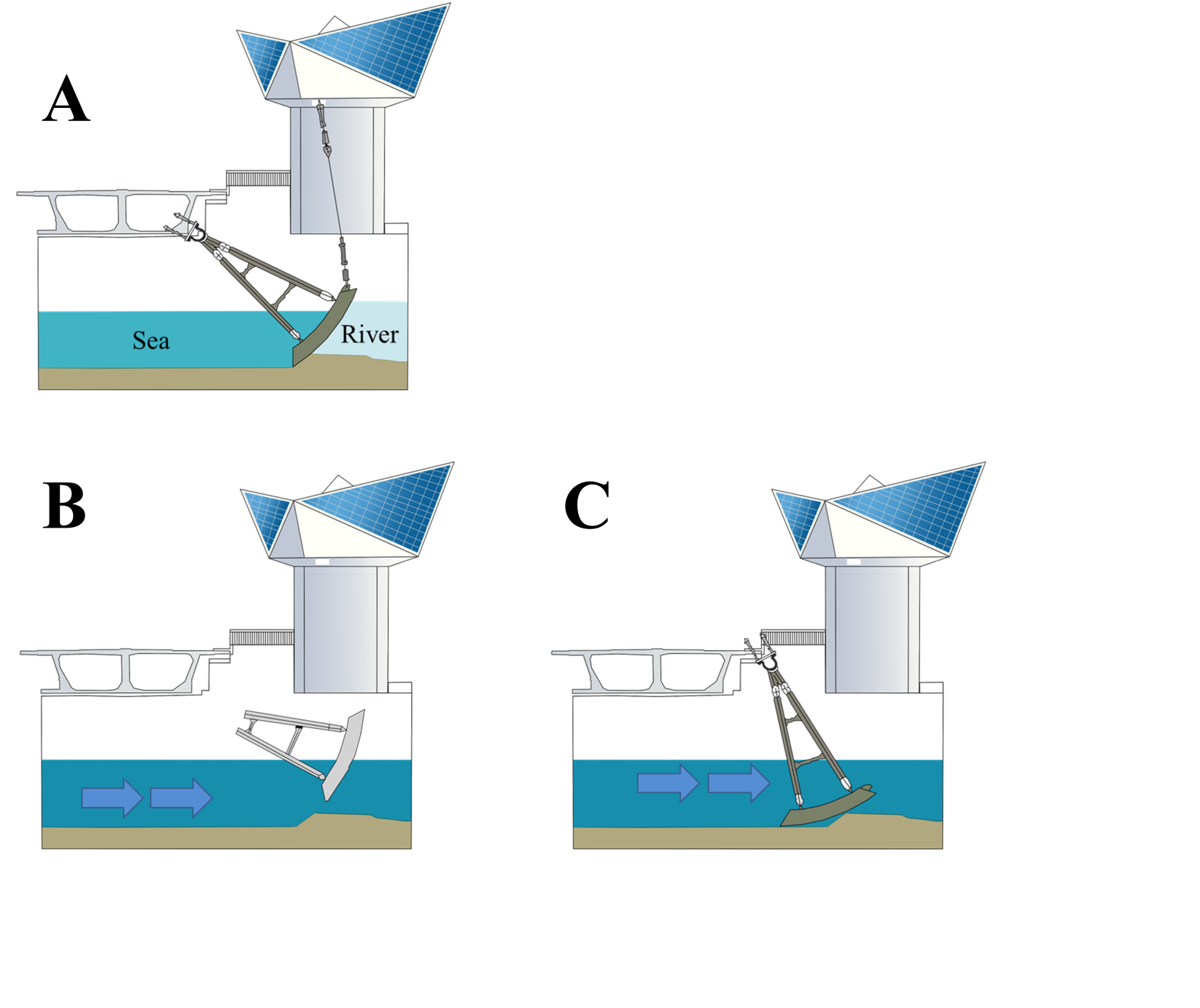

Supplement: S1 Fig — (A) The regulatory gate remains closed, blocking freshwater and seawater mixing. (B) The underflow and (C) the overflow method for opening the regulatory gate. (TIF) [file pone.0328372.s001.tif]

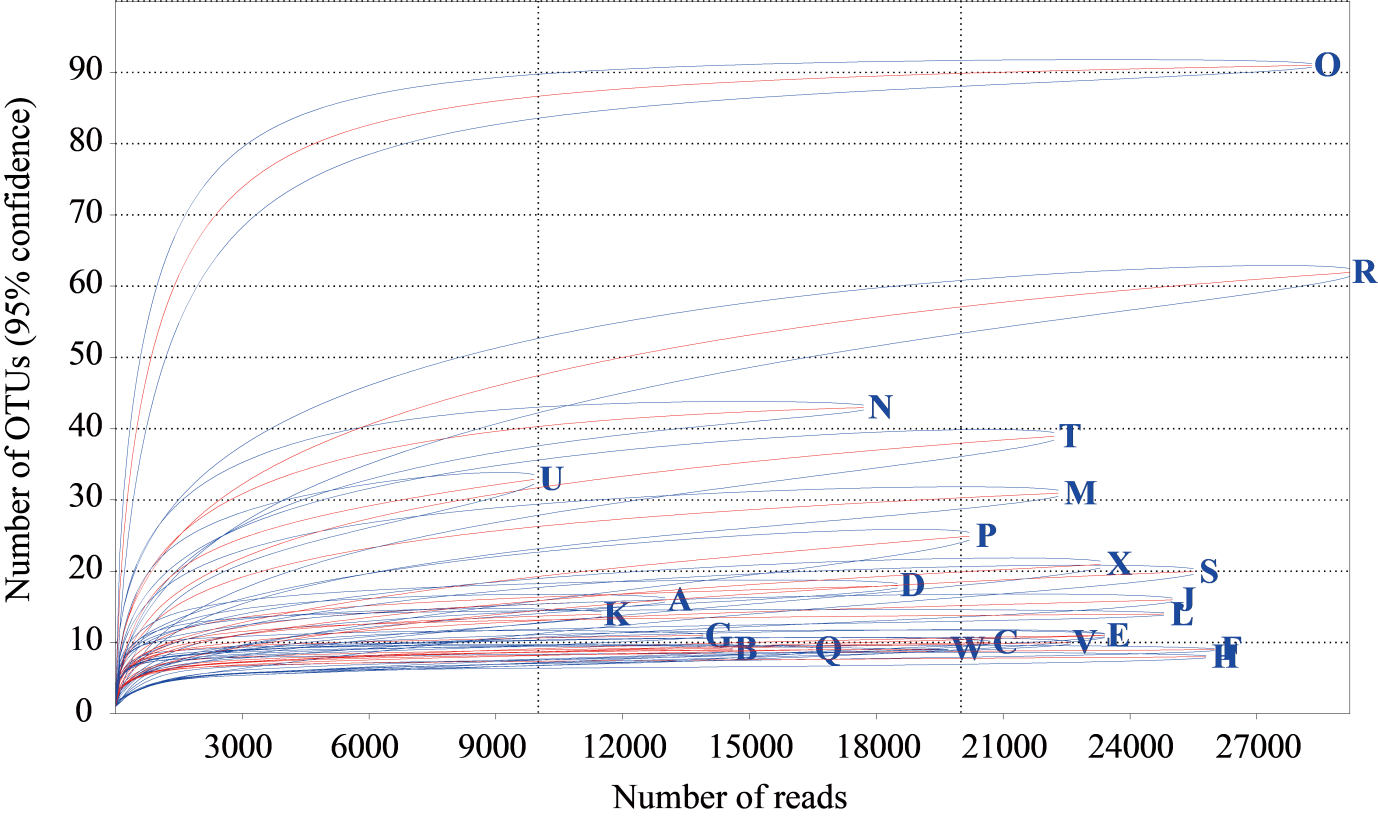

Supplement: S2 Fig — (A to X: Sample 1 to Sample 24). (TIF) [file pone.0328372.s002.tif]

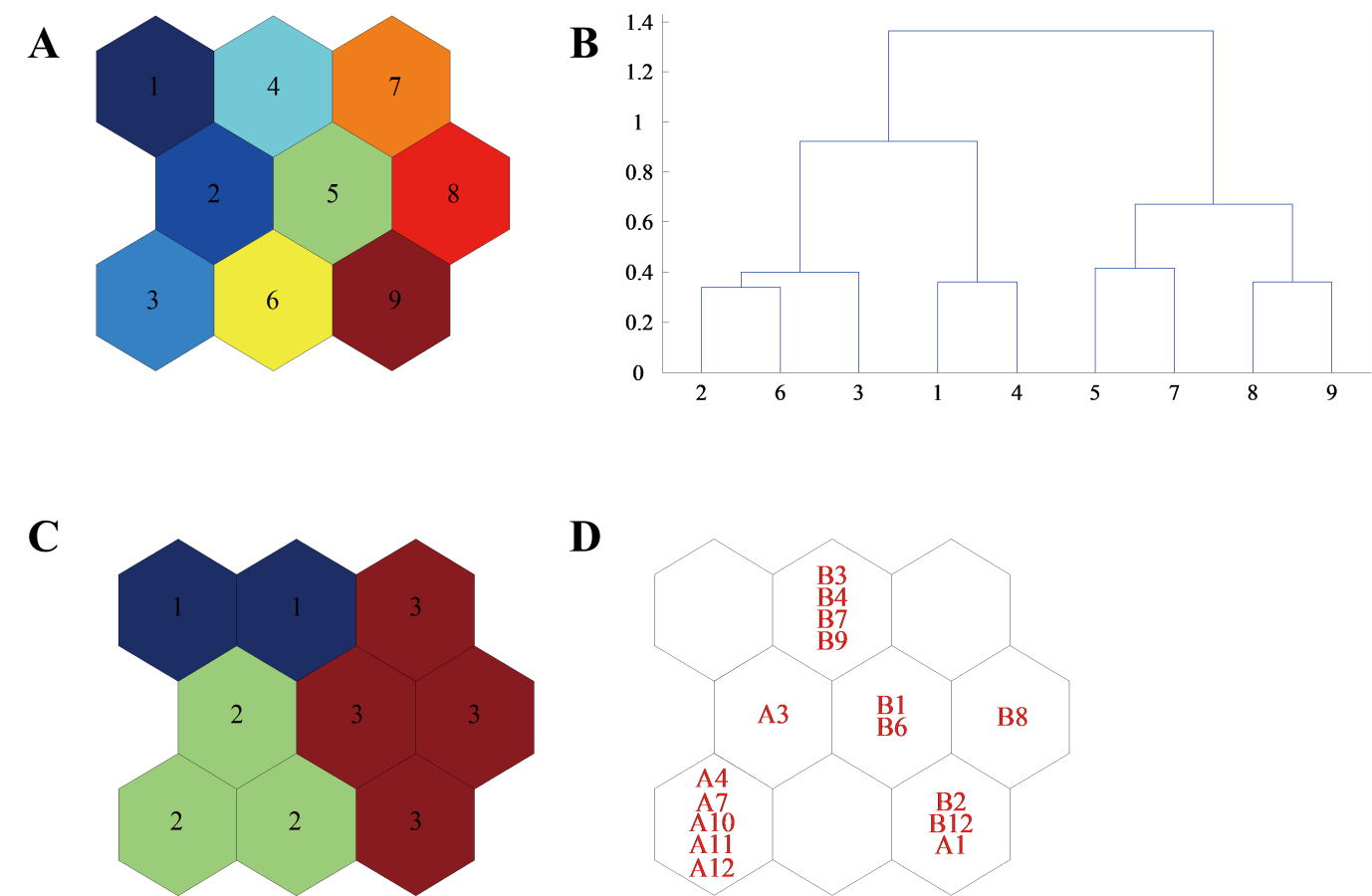

Supplement: S3 Fig — (A) Clustering of the cells on the map plane, (B) dendrogram showing dissimilarity of the cells in the map, (C) Clustering of the cells on the map plane by three groups and (D) The patterning results for before and after NRE opening on the SOM plane, B: Before NRE opening; A: After NRE opening. (TIF) [file pone.0328372.s003.tif]

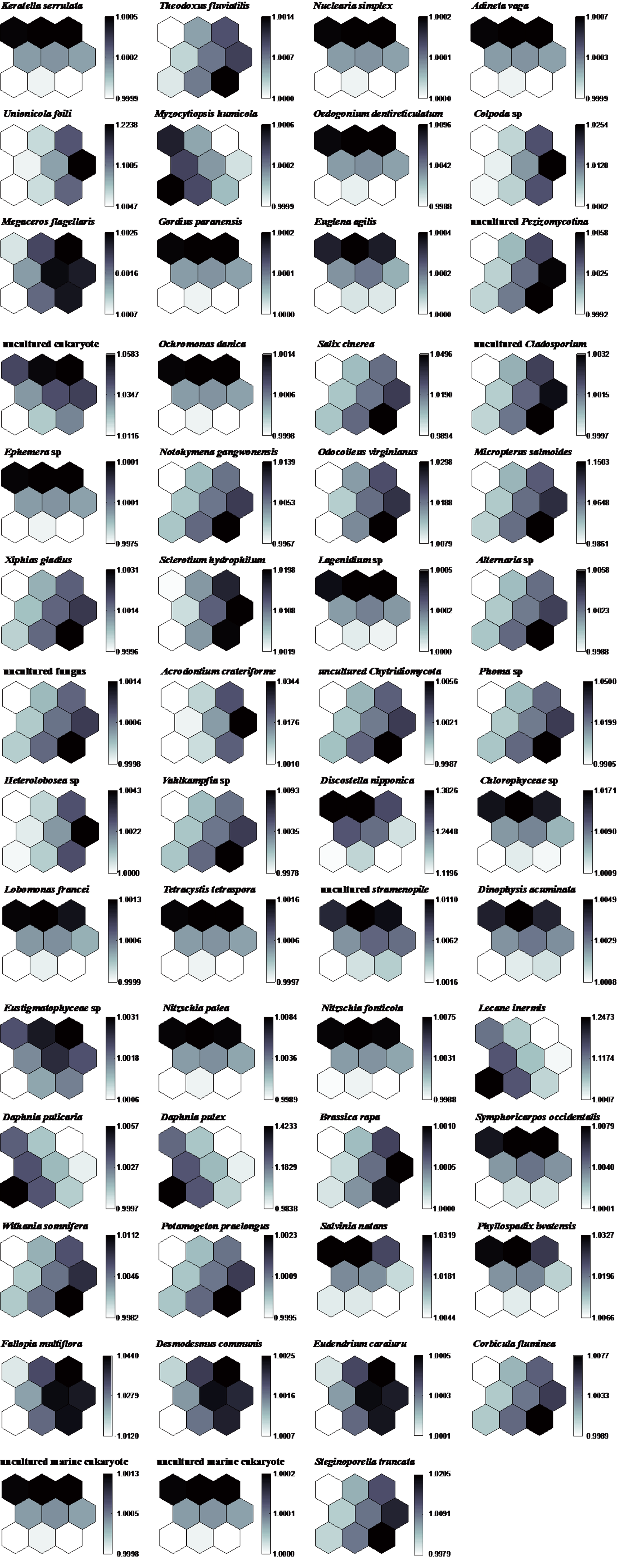

Supplement: S4 Fig — Colour scale is related to distances between map units. Black colours represent large distances and white colours represent small distances. (TIF) [file pone.0328372.s004.tif]
